# Editorial: Reviews in psychology of language

**DOI:** 10.3389/fpsyg.2025.1569614

**Published:** 2025-02-25

**Authors:** Antonio Benítez-Burraco, Antonio Bova, Thomas L. Spalding

**Affiliations:** ^1^Department of Spanish, Linguistics, and Theory of Literature, Faculty of Philology, University of Seville, Seville, Spain; ^2^Department of Psychology, Faculty of Psychology, Università Cattolica del Sacro Cuore, Milan, Italy; ^3^Department of Psychology, Faculty of Arts, University of Alberta, Edmonton, AB, Canada

**Keywords:** psycholinguistics, cognition, language acquisition, second language learning, language disorders, language evolution

Traditionally, research on language facts has focused on overt linguistic behaviors. Linguists have examined human languages to learn about their fundamental components and how these basic pieces are arranged into more complex sets, from syllables to words to sentences to discourses. Typologists have described hundreds of languages and found that they share a core set of components and structural principles, supporting the view that all human languages are similarly designed and fulfill similar roles equally well. Dialectologists and sociolinguists have characterized different varieties of each single language and show that intralinguistic variation follows similar paths and results from similar triggering factors as interlinguistic diversity. And the same is true for language change as characterized by historical linguistics. Nonetheless, for a long time, languages were regarded as cultural artifacts mostly, like food practices, religions, or types of costumes. In the second half of the twentieth century, insights on how languages are acquired by children started to change this traditional conceptualization of languages (and of language as a human distinctive trait). Nowadays, language is generally construed as a key component of the human phenotype, particularly, of our mind/brain. Nativist views of language gained preeminence during the last decades, to the extent that language was even thought of as an organ that grows in our brain under genetic guidance. This view has been toned down, so that both our genome and our environment are thought to contribute to our distinctive linguisticality. In any case, it is generally acknowledged that if we want to understand the ultimate nature of language, it is necessary to delve into the brain black box in order to know which aspects of our mind/brain support language and in particular, if they are specific to language or domain-general by nature.

Two related disciplines have led such a crucial line of inquiry: psycholinguistics and neurolinguistics. The former aims to know about the mental processes that allow us to understand and produce language, but also to acquire our mother tongue and to learn other languages. The latter tries to identify and characterize the brain circuits that support language processing and language acquisition/learning. In other words, psycholinguistics is mostly concerned with the software of language, whereas neurolinguistics is mostly interested in its hardware. Over the years, as with linguistics more generally, psycholinguistics has evolved to be more and more methodologically complex and theoretically diverse. Many technological advances (e.g. eye-tracking) allow psycholinguists to conduct truly sophisticated experiments to address questions previously impossible to answer. Similarly, different theories about language processing and acquisition have emerged with time, and research has become increasingly diverse, as non-European languages and non-standard varieties of languages have been examined by psycholinguists. Finally, research has also evolved to be more multidisciplinary, as contacts with other subfields of linguistics (particularly, neurolinguistics), and other disciplines (like computational science, or biology) are helping psycholinguists to construct more robust hypotheses about the nature of language and to explore new avenues of research.

The aim of this Research Topic is to gather comprehensive and up to date review articles on key aspects of psycholinguistics. Because psycholinguistics is a notably dynamic and increasingly complex field, as noted, it is difficult (and urgent) for researchers to be up to date. In this Research Topic, we have brought together 10 contributions from 30 scholars.

Starting with articles addressing basic aspects of psycholinguistic research, the article authored by Sun and Lin provides a state-of-the-art review of past research on metonymy, a core cognitive operation in language processing, acquisition, and change. The bibliometric analysis performed by these authors reveals that theoretical and cognitive issues are still at the forefront of research on this topic, but also that some underexplored aspects are gaining attention, particularly, the intersection between metonymy and other diverse domains, including the emotional sphere, selected social and cultural dimensions, and other communicative modalities, particularly, vision. In turn, Renström reviews the potential impact of pronoun usage on gender conceptualization, with some attention to attitudinal issues, to understand both the resistance and the promotion of gender-inclusive language and linguistic gender reforms, more generally.

Reflecting ample interest, both past and present, in the psycholinguistics of language change during the lifespan, but also of language disorders, we have included 3 contributions on these issues in the Research Topic. Ansari et al. have conducted a meta-analysis of recent literature about word learning by children with Developmental Language Disorder (DLD), which contributes to clarify key features of the process, but also to suggest ways of improving the interventions aimed to facilitate vocabulary learning by these children. Janssen et al. discuss the role of executive function in the (dis)abilities of children with DLD for storytelling, also with the ultimate objective of achieving better strategies to ameliorate their reduced narrative capacities. Finally, Lesecq et al., which also focus on non-typical children, have found that gifted children exhibit more heterogeneous reading abilities compared to their peers.

For a long time, the field of psycholinguistics has been highly interested in the mechanisms involved in second language learning. Two of the articles comprising the Research Topic address this issue. Zhang et al. examine the impact of parents' investment behavior on the learning success of a sample of Chinese students of English as a second language (L2). They found that both participation and investment by the parents have a positive effect on the motivation and learning behavior of children. In turn, Hui and Chen provide insights on the effect of socioeconomic status on pragmatic awareness, using a sample of Chinese students learning English as an L2.

Two additional contributions to the Research Topic examine aspects of the psycholinguistics of language acquisition/learning, but with a focus on methodological issues. Harrag et al. have authored a article evaluating the utility of semantic indices as diagnostic and assessment tools of language change. Specifically, they focus on the role of propositional density, i.e. the amount of information conveyed per language segment, as a reliable tool for tracking language change during aging. With regards to the article by Li and Zhong, it reviews the usage of eye-tracking techniques for gaining insights on the psycholinguistics of translation. Their review article highlights several domains to which eye-tracking has been successfully applied, mostly related to human-machine interaction, as well as ongoing trends in the psycholinguistics of translation, which seems to be evolving to be more empirical and multidisciplinary, but less theoretically-motivated with time.

Finally, the article by Benítez-Burraco provides a general framework for language evolution studies in the human species. According to the author, modern languages resulted from selected changes in our cognition, including the emergence of improved ways of processing grammar rules. These changes might have resulted in part from our trend toward more prosocial behaviors, which also fostered the cultural evolution of languages. Ultimately, the article argues for a multidisciplinary approach to language evolution in which psycholinguistic research would still play a key role.

